# Preliminary in vitro cytotoxic evaluation of *Uncaria gambier* (Hunt) Roxb extract as a potential herbal-based pulpotomy medicament

**DOI:** 10.1186/s12906-023-04163-w

**Published:** 2023-09-20

**Authors:** Bee Chin Tan, Alida Mahyuddin, S. Nagarajan M. P. Sockalingam, Ahmad Shuhud Irfani Zakaria

**Affiliations:** Department of Family Oral Health, The National University of Malaysia, Jalan Raja Muda Abdul Aziz, Kuala Lumpur, 50300 Malaysia

**Keywords:** Mineral trioxide aggregate, Pulpotomy, Toxicity, Transmission electron microscopy, *Uncaria gambir* extract

## Abstract

**Background:**

The downfall of formocresol as a pulpotomy medicament highlights the importance of cytotoxic evaluation and the establishment of a safe concentration of dental material prior to its usage in the oral cavity. *Uncaria gambir* is an herbal plant that possesses antimicrobial, anti-inflammatory and antioxidant properties, suggesting its potential as an alternative medicament for pulpotomy. However, there are not many studies published on its cytotoxicity, with some using non-standardised techniques and reported variable outcomes. Here, we investigated the concentration and time-dependent toxicity of *Uncaria gambir* extract towards the M3CT3-E1 cell line and compared it with the gold standard pulpotomy medicament: mineral trioxide aggregate (MTA).

**Methods:**

*Uncaria gambir* extracts at concentrations ranging from 1000 to 7.8 µg/ml and MTA eluates at 4- and 48 h setting times were prepared. 10% dimethyl sulfoxide (DMSO) and culture media were used as positive and negative controls respectively. Cell viability on days 1, 2, 3 and 7 was analysed using Alamar Blue and Live and Dead Cell assay. Any morphological cellular changes were evaluated using transmission electron microscopes (TEM). Data were analysed using a two-way mixed Analysis of Variance (ANOVA).

**Results:**

The interaction between the concentration and exposure time on the fluorescence intensity of *Uncaria gambir* extract and MTA 48 h was found to be statistically significant (*p* < 0.001). No cytotoxic effects on the cells were exerted by both MTA 48 h and *Uncaria gambir* extract at a concentration below 500 µg/mL. TEM analysis and Live and Dead Cell assay for both materials were comparable to the negative control. No significant differences in fluorescent intensity were observed between *Uncaria gambir* extract at 500 µg/mL and MTA 48 h (*p* > 0.05).

**Conclusion:**

*Uncaria gambir* extracts at a maximum concentration of 500 μg/mL are non-cytotoxic over time and are comparable to the MTA.

## Background

Pulpotomy is a dental procedure which involves the removal of the infected coronal pulp tissue while maintaining the integrity of the radicular pulp tissue intact [1]. Following removal of the infected and inflamed coronal pulp, a medicament is placed over the amputated pulp tissue. This medicament will react with the vital radicular pulp tissue, forming a mechanical barrier that protects the radicular pulp and wall-off the pulp tissue from future bacterial invasion. In addition, some of the pulpotomy medicament also exerted antibacterial effects which enhanced the success rate of the procedure.

Over the years, various materials have been proposed and used as pulpotomy medicament. Historically, formocresol was the material of choice but its usage has been discontinued following the risk of nasopharyngeal cancer in humans [2]. This has led to the work on searching for new material as an alternative to formocresol, with a similar success rate and most importantly, safe. Non-setting calcium hydroxide was later used as pulpotomy medicament due to its excellent antibacterial action and ability to induce calcific barrier formation at the site of tissue amputation [3]. Despite the formation of a calcific barrier, the outcome of non-setting calcium hydroxide in pulpotomised teeth was poor, with a success rate of less than 60% [4]. This failure is associated with the formation of a ‘tunnel’ within the calcific barrier, caused by the entrapment of blood vessels and collagen bundles during the slow process of the barrier formation [3]. Further, long-term usage of the material causes desiccation of the dentinal protein and collapse of the collagenous framework, leading to internal resorption and subsequent root fracture [5].

Adopting a similar concept of non-setting calcium hydroxide, mineral trioxide aggregate (MTA) was introduced into the market by Mahmoud Torabinejad in the early 1990s. The main constituents of MTA are tricalcium silicate, dicalcium silicate, tricalcium aluminate and bismuth oxide [6]. MTA has a pH of 12.5, which contributes to its antibacterial activity. When placed in the cavity, in the presence of moisture, the component of MTA will dissociate and release calcium ions. This ion will interact with the tissue phosphate to form a hydroxyapatite crystal on its surface. It also promotes cellular attachment and proliferation and induces modulation of cytokine production which encourages the hard tissue-forming cells to differentiate and migrate. This leads to the formation of a hard tissue calcific barrier, which protects the radicular pulp [6, 7]. The success rate of MTA pulpotomy was reported to be between 96% to 100% [7], and it has been regarded as the gold standard of pulpotomy medicament ever since. However, the material is associated with tooth discolouration and is costly, which may affect the overall treatment cost [8]. The value of using MTA as the material of choice for pulpotomy medicament is also being questioned as the deciduous teeth will be replaced by their permanent successor later. Thus, the quest searching for an ideal pulpotomy medicament is still actively carried out by researchers worldwide.

The extract from natural products has come into sight as these products are readily available, exert minimal toxicity and are cheap [9]. The extract of *Uncaria gambier (Hunt) Roxb* or *Uncaria gambir* has caught our attention, mainly due to its reported anti-inflammatory, antibacterial, antioxidant and wound healing capabilities [10]. *Uncaria gambir* plant grows primarily in areas with a tropical climate, such as South America, Africa and South-East Asia [11]. The extract has been used as traditional medicine for centuries as remedies for burns, headaches, diarrhoea, cancer sores, sore throat gargles, dental pain and swollen gums [12, 13]. Further, the extract of *Uncaria gambir* was declared an official pharmacopoeia medicine in Japan [11]. The therapeutic effects of *Uncaria gambir* extract highlight its potential as a candidate for alternative natural-based pulpotomy medicament.

The cytotoxic evaluation and the establishment of a safe concentration of a dental material prior to its usage in the oral cavity are of paramount importance. However, study on the cytotoxicity of the *Uncaria gambir* extract is scarce, with variable outcomes and using non-standardised methods. Therefore, the present study was conducted to investigate the bio-compatibility of *Uncaria gambir* extracts on MC3T3-E1 cells in vitro by evaluating the impact of different concentrations and exposure times on its cytotoxicity. Further, the value of the extract as a potential pulpotomy medicament is tested by comparing the cytotoxicity of the extract with MTA, the gold standard pulpotomy medicament.

## Methods

### Preparation of test materials

The *Uncaria gambir* leaves were collected from Bukit Diman, Ajil, Terengganu, Malaysia by an expert from the Malaysian Agricultural Research and Development Institute (MARDI). Later, a voucher specimen number representing the *Uncaria gambir* (HTBP 4320) was deposited following verification of the samples by the botanist at Herbarium Putrajaya Botanical Garden, Putrajaya, Malaysia. The cultivation, handling and processing of the plant were carried out in accordance with the Malaysian Standard on Good Agricultural Practice (GAP) – Part 8: Herbs [14] and the Malaysian Standard on Good Agricultural Practise (GAP) – Crop commodities [15].

The leaves were cleaned thoroughly from any dirt and were air-dried at room temperature. The dried leaves were ground into a fine powder, weighed (Mettler, Toledo) at 3.1 kg and macerated with methanol (Merck, Germany). The maceration process was done by placing the powdered *Uncaria gambir* in a container before pouring the methanol over the powder until it covered the material completely. The container was kept closed for 72 h at room temperature and was stirred periodically to ensure complete extraction. Upon completion of the maceration process, the methanol was removed through evaporation under reduced pressure using a rotary evaporator (Rotavapor^®^ R-210, Buchi Labortechnik, AG) yielding 127.37 g of the crude extract of *Uncaria gambir*.

The crude extract was dissolved in 1% DMSO (Merck, Germany) at a concentration of 2000 μg/mL using the test tube shaker (Ingenieurbüro CAT, M.Zipperer GmbH, Germany). DMSO was used as a solvent to promote the solubilisation of the extract in culture media. The amphipathic property of DMSO favours the dissolution of the *Uncaria gambir* extract which possesses both polar and nonpolar components. Later, the mixture was filtered through a 0.2 μm syringe filter (Sartorius Stedim, Germany) to obtain a purified *Uncaria gambir* extract [16]. Subsequently, the purified extract underwent a 2-fold serial dilution into 8 different concentrations ranging from 1000 μg/mL of the extract in 0.5% DMSO to 7.8 ug/mL of extract in 0.0039% DMSO. The maximum concentration was selected based on our pilot study and a previous study done by Nur Sazwi et al. [17].

ProRoot^®^ MTA (Dentsply Tulsa Dental Specialties, USA) eluates were prepared in two different setting times: 4 and 48 h (MTA 4h and MTA 48h respectively), based on the manufacturer's instruction. Under the biosafety cabinet, 1 sachet of MTA powder and 1 ampule of liquid at a 1:1 ratio were dispensed on the mixing pad and mixed thoroughly for 1 minute until all the powder particles were hydrated. The freshly mixed MTA was placed into a silicon mould measured 8 mm x 2 mm and was left to set at 4 and 48 h in an incubator (Thermo Scientific, USA) at 37°C. Once set, the MTA 4h and 48h were separated from the silicon mould and immediately immersed in the culture media for 24 h at 37°C with 5% carbon dioxide (CO_2_). The extraction ratio of the specimen surface area was 1.25 cm^2^/mL, following the standard ISO 10993-12 [18]. Subsequently, the MTA eluates were filtered using a 0.2 μm syringe filter [19, 20].

### Cell lines preparation and culture

The mouse fibroblast cell lines (MC3T3-E1) were obtained from the institution’s bank cell. The cells were cultured in Dulbecco’s Modified Eagle Media (DMEM) (Thermo Fisher Scientific, USA) and supplemented with 10% fetal calf serum (Sigma-Aldrich, Germany), 1% penicillin/streptomycin (Thermo Fisher Scientific, USA) and 1% L-glutamate (Thermo Fisher Scientific, USA) at 37°C and 5% CO_2_. The cells were passaged every 3 days based on the standard trypsin/ethylenediaminetetraacetic acid (EDTA) protocol according to Freshney [21]. Cells between the fourth and sixth passages were used in this study.

**Fig. 1:**
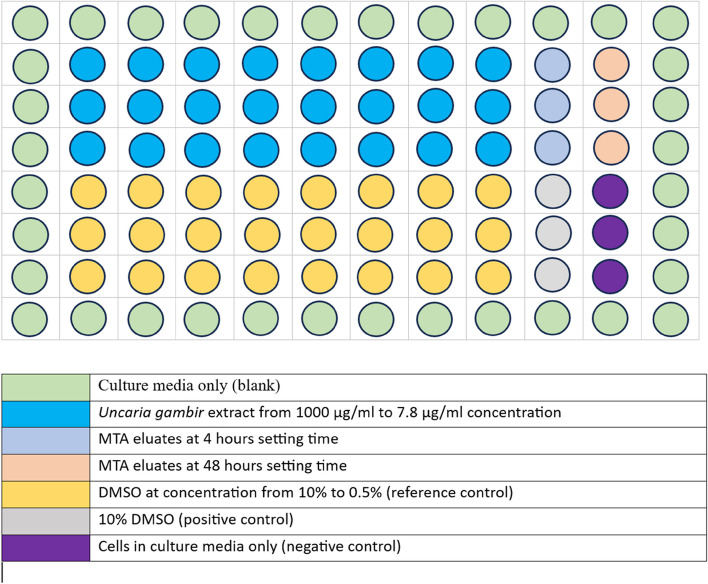
The layout of the 96-well plate for both test materials and control solutions. Note that each of the material/solution was done in triplicates

The cells at a density of 5 x 10^3^ in 100 uL of culture media were seeded in wells of a 96-well plate (Biologix, USA). The 96-well plate was incubated at 37°C, 5% CO_2_ for 24 h to obtain a semiconfluent layer of cells in each well. After 24 h of incubation, the culture media in each well was discarded. 100 uL of the *Uncaria gambir* extract in eight different concentrations, ranging from 1000 μg/mL to 7.8 μg/mL, MTA eluates at 4- and 48-h setting time, positive and negative control solution, and DMSO with concentrations ranging from 0.0039% to 0.5% (reference control) were added into 96-well plates in triplicates (technical replicates) according to their respective group. 10% of DMSO and culture media were used as positive and negative control respectively (Fig. 1). Following the addition of test materials and control solutions, the well plates were incubated at 37°C with 5% CO_2_ for 1, 2, 3, and 7 days respectively before the commencement of the cytotoxicity tests.

### Alamar Blue cytotoxic assay

Following the respective culture time of 1, 2, 3, and 7 days, 10 μL of Resazurin dye (Sigma-Aldrich, Germany) was added to the well plate and mixed gently in an incubator shaker (IKA, Germany) to ensure a homogeneous distribution of the dye. Then, the well plate was incubated at 37°C with 5% CO_2_ for 2 h and 30 minutes. The amount of the resorufin produced by the viable cells is reflected by the increase in the fluorescence intensity. This was measured by monitoring the increase in fluorescence intensity at an emission and excitation wavelength of 590 nm and 560 nm respectively using the 96-well plate reader (Thermo Scientific, USA). The test was repeated three times, *n* = 3 using different well plates (biological replicates) and the mean percentage of the cell viability was quantified in line with the standard ISO 10993-5 [22].

### Live and Dead Cell assay

The Live and Dead dye solution (Abcam, USA) was prepared based on the manufacturer's instructions. 5 μL of 1000x Live and Dead dye was diluted with 1 mL of phosphate-buffered saline (Thermo Fisher Scientific, USA) and underwent 200-fold dilution to achieve 5x Live and Dead dye. Following the indicated culture time of 1, 2, 3, and 7 days respectively, the culture media in the 96-well plate was discarded. 200 μL of 5x Live and Dead dye was added to each well followed by incubation of the well plate for 10 min at room temperature prior to examination under the inverted fluorescence microscope (Olympus, Japan).

### TEM analysis

TEM analyses were conducted to analyse the potential morphological changes in the cells following exposure to the test material and control solutions. Following confirmation of the cytotoxic concentration of *Uncaria gambir* extract, samples consisting of positive and negative control, MTA 4h and 48h and *Uncaria gambir* extract at 500 µg/mL concentration were prepared. The cells co-cultured with positive control and MTA 4h were analysed following 24 h of exposure only, while cells exposed to the negative control, MTA 48h and 500 µg/mL of *Uncaria gambir* extract were analysed following 1 and 7 days of exposure to the test material.

The M3CT3-E1 cells were prepared and cultured in a 24-well plate (Biologix, USA) as described earlier, with 2.5x10^4^ cells seeded per well while 400 μL of culture media/test material was used in each well accordingly. After the respective 1 and 7 days of incubation, the preparation of TEM samples commenced. The samples were prepared based on the protocol described by IHC World [23], where the cells underwent various fixation and embedding processes, resulting in the formation of a cell pellet embedded in a resinous block.

The initial thick sections of the resin block measuring between 0.5 to 1 µm were stained with toluidine (EMS, USA). Next, using an ultramicrotome (Leica, Germany) the ultrathin sections of resin block at 60 to 90 nm thick were prepared. Upon completion, the sections were collected on grids and were stained with uranyl acetate and lead citrate (BDH, UAE) for 15 and 5 min respectively. Finally, the cell samples were viewed under TEM (Philips CM12 120kV, The Netherlands) and the ultrastructure and morphology of cells were assessed [24].

### Data analysis

The collected data were analysed using the Statistical Package for the Social Sciences (SPSS) version 22 (IBM, Armonk, NY, USA). The mean percentage of cell viability for the test material and control solutions at different time intervals was calculated through the percentage difference between the treated and untreated control cells after corrected with blank control by using the following formula [25]:

$$\mathrm{The\;mean\;percentage\;of\;cell\;viability }\left(\mathrm{\%}\right)=\frac{(\mathrm{FI }590\mathrm{\;of\;test\;agent }-\mathrm{ FI }590\mathrm{\;of\;blank\;control})}{(\mathrm{FI }590\mathrm{\;of\;untreated\;control }-\mathrm{ FI }590\mathrm{\;of\;blank\;control})}\times 100$$

where, FI 590 = the mean value of the measured fluorescence intensity at 590 nm emission (560 nm excitation) and blank control = well that contained culture medium only.

The Two-way Mixed Analysis of Variance (Two-way Mixed ANOVA) test was used to compare the fluorescence intensity of different concentrations of *Uncaria gambir* extract, positive control and MTA eluates against the negative control at different exposure times. A *p*-value of less than 0.05 was considered statistically significant.

## Results

### Cytotoxicity of the solvent dimethyl sulfoxide (DMSO)

DMSO at high concentrations is cytotoxic to cells and able to induce cell death and apoptosis. 10% DMSO was used as a positive control in this study. However, during the preparation of the *Uncaria gambir* extract, DMSO was used as a solvent, with the highest concentration used at 0.5%. Alamar Blue assay was performed to evaluate the cytotoxic potential of DMSO used as a solvent in the present study. The mean percentage of cell viability was over 94% for all concentrations tested. No significant difference was observed between the DMSO at all concentrations and negative control as shown by the Kruskal-Wallis H test over the 7 days study period, with *p* > 0.05 (Table 1). Thus, DMSO at a concentration below 0.5% is suitable to be used as a solvent for the preparation of *Uncaria gambir* extract and did not exert any toxic effect on the M3CT3-E1 cell lines.

**Table 1:** Mean percentage of cell viability of DMSO at different concentrations

**Concentration of *Uncaria gambir* extract (µg/mL)**	**Concentration of corresponding DMSO (%)**	**Mean percentage of cell viability (%)± SEM**			
		**Day 1**	**Day 2**	**Day 3**	**Day 7**
1000	0.5	94.53	95.34	98.45	98.08
500	0.25	94.96	96.06	98.20	98.15
250	0.125	96.04	96.29	98.48	98.24
125	0.063	95.73	96.72	99.43	98.32
62.5	0.031	99.49	96.82	96.00	98.58
31.25	0.016	99.64	97.12	99.10	98.87
15.6	0.008	98.45	98.68	98.03	99.35
7.8	0.004	99.27	98.71	100.04	99.80
	*p*-value^1^	0.921	0.717	0.278	0.751

*SEM* Standard error of the mean

^1^*p* is significant at 0.05

### Alamar blue assay

**Fig. 2:**
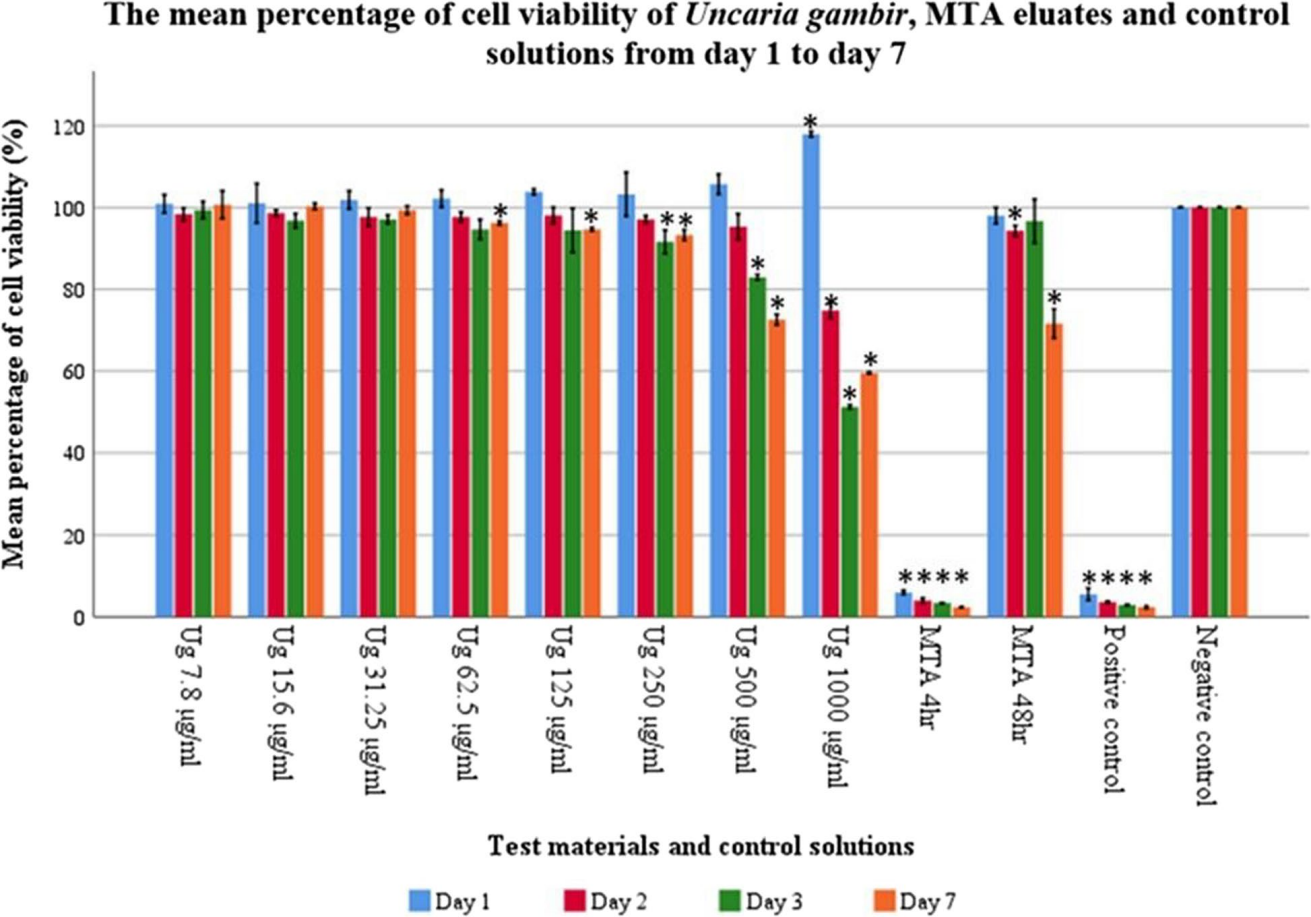
The mean percentage of cell viability of *Uncaria gambir* extracts, MTA eluates and control solutions from day one to day seven. The asterisk indicates significant differences to the negative control (*p* < 0.05)

The cytotoxicity of the test materials and control solutions were evaluated using the Alamar Blue assay, with the minimum threshold of 70% regarded as non-cytotoxic, in line with the ISO 10993-5 standard [22]. On day 1, 100% of the mean percentage of cell viability was observed for MTA 48h and *Uncaria gambir* extract at all concentrations. MTA 4h was cytotoxic to cells with a mean percentage of cell viability of 5.9%. From day 2 onwards, the mean percentage of cell viability declined over time for MTA 48h and *Uncaria gambir* extract at 1000 and 500 µg/mL concentration. Both MTA 48h and the extract at 500 µg/mL were non-cytotoxic to the cells at day 7, with the mean percentage of cell viability exceeding 70%. However, *Uncaria gambir* extract at 1000 µg/mL was cytotoxic to the cells on days 3 and 7 of exposure. The mean percentage of cell viability of the extract at a concentration below 250 µg/mL was constantly beyond 90% throughout the study period (Fig. 2).

### The effects of different concentrations and exposure time on the cytotoxicity of Uncaria gambir extracts and MTA eluates

Different concentrations and exposure times may influence the cytotoxicity of MTA and the *Uncaria gambir* extract, which can be determined based on the fluorescence intensity. The material is regarded as non-cytotoxic if it produces high fluorescence intensity and vice versa. Two-way mixed ANOVA with post hoc multiple and pairwise comparison tests was conducted to test this relationship. Multiple comparisons with the Games-Howell post hoc test were performed to evaluate the effects of MTA eluates and *Uncaria gambir* extract at a specific concentration against the negative control. A pairwise comparison with Bonferroni adjustment was conducted to determine the differences in fluorescence intensity over a time span for each test material.

MTA 4h consistently showed significantly lower fluorescence intensity throughout the 7 days (*p* < 0.001) suggesting the toxic nature of the material. MTA 48h and *Uncaria gambir* extract at 500 µg/mL showed comparable fluorescence intensity with negative control except on days 7 and days 3 and 7 respectively, where a significant reduction of fluorescence intensity was observed ( *p* ≤ 0.001). *Uncaria gambir* extract at 1000 µg/mL exhibited a significant increase in fluorescence intensity on day 1 but reduced significantly on day 2 onwards when compared to the negative control. No significant difference in fluorescence intensity was detected for *Uncaria gambir* extract at a concentration below 250 µg/mL (Table 2).

**Table 2:** Multiple comparisons of fluorescence intensity of test materials with negative control and pairwise comparisons of fluorescence intensity of test materials and controls over seven days

**Time (day)**		**Test materials and controls**											
		**1000 μg/ml Ug**	**500 μg/ml Ug**	**250 μg/ml Ug**	**125 μg/ml Ug**	**62.5 μg/ml Ug**	**31.25 μg/ml Ug**	**15.6 μg/ml Ug**	**7.8 μg/ml Ug**	**MTA 48 h**	**MTA 4 h**	**Positive control**	**Negative control**
1	FI (mean ± SD)	521.78 ± 4.09^a^	467.76 ± 18.08^d^	457.04 ± 32.80^g^	459.38 ± 9.40^j^	452.18 ± 16.69^m^	450.78 ± 17.21^p^	447.28 ± 28.90^t^	446.48 ± 16.90^x^	433.74 ± 15.13^B^	26.50 ± 2.77^E^	24.56 ± 7.64	442.32 ± 6.16^F^
	Mean cell viability (%)	117.96	105.75	103.33	103.86	102.23	101.91	101.12	100.94	98.06	5.99	5.55	100.00
	*p*-value	< 0.001	0.320	0.988	0.179	0.959	0.986	1.000	1.000	0.970	< 0.001	< 0.001	-
2	FI (mean ± SD)	592.10 ± 7.87^b^	755.96 ± 57.09^e^	769.24 ± 31.09^h^	766.58 ± 16.13^k^	774.22 ± 39.93^n^	773.22 ± 13.29^q^	781.44 ± 33.75^u^	778.74 ± 22.16^y^	746.7 ± 21.88^C^	31.80 ± 6.71	28.62 ± 3.19	792.04 ± 33.28^G^
	Mean cell viability (%)	74.76	95.44	97.12	98.05	97.75	97.62	98.66	98.32	94.28	4.01	3.61	100.00
	*p*-value	0.001	0.967	0.983	0.994	0.999	0.970	1.000	0.999	0.433	< 0.001	< 0.001	-
3	FI (mean ± SD)	535.34 ± 10.07^a^	866.08 ± 12.69^f^	956.96 ± 42.35^i^	986.56 ± 72.05^l^	988.56 ± 37.94^o^	1013.56 ± 21.29^r^	1010.96 ± 30.11^v^	1037.76 ± 33.76^z^	1010.36 ± 73.17^D^	35.42 ± 1.04^F^	30.40 ± 1.96	1043.96 ± 11.76^H^
	Mean cell viability (%)	51.28	82.96	91.67	94.50	94.69	97.09	96.84	99.41	96.78	3.39	2.01	100.00
	*p*-value	< 0.001	< 0.001	0.101	0.791	0.288	0.349	0.563	1.000	0.986	< 0.001	< 0.001	-
7	FI (mean ± SD)	644.42 ± 5.40^c^	785.60 ± 23.02^e^	1009.16 ± 21.38^i^	1024.56 ± 7.40^l^	1041.56 ± 5.59^o^	1075.16 ± 20.33^s^	1085.36 ± 16.95^w^	1089.76 ± 48.84^A^	775.24 ± 49.66^C^	24.76 ± 1.30^E^	25.38 ± 4.19	1081.96 ± 9.73^I^
	Mean cell viability (%)	59.56	72.61	93.27	94.69	96.27	99.37	100.31	100.72	71.65	2.29	2.35	100.00
	*p*-value	< 0.001	< 0.001	0.011	< 0.001	0.003	1.000	1.000	1.000	0.001	< 0.001	< 0.001	-

Multiple comparisons with Games-Howell post hoc test for comparison of test materials with negative control, *p* is statistically significant at α = 0.05

*FI* Fluorescence intensity, *SD* Standard deviation, *Ug Uncaria gambir*, *MTA* Mineral trioxide aggregate

Pairwise comparisons with Bonferroni adjustment for comparison of test material at particular concentration over time; different letters (a vs b) in each column indicated significant different for test material at specific concentration over time (*p* < 0.05) while similar letters (a vs a) denote insignificant difference (*p* > 0.05)

The effect of exposure time on the fluorescent intensity of different test materials and control solutions showed a significant difference between day 7 and day 1 ( *p* < 0.05) except for MTA 4h and positive control, where no significant difference in fluorescence intensity was observed as represented by the vertical column of Table 2.

Comparison of fluorescence intensity between MTA 48h and *Uncaria gambir* extract showed no statistically significant difference at a concentration below 500 µg/mL throughout the study period except on day 7 where *Uncaria gambir* extract at a concentration below 250 µg/mL showed a significantly higher fluorescence intensity over MTA 48h (*p* < 0.05). *Uncaria gambir* extract at 1000 ug/mL concentration on the other hand showed significantly lower intensity than MTA 48h through the study period ( *p* < 0.001) (Table 3).

**Table 3:** Multiple comparisons of fluorescence intensity of *Uncaria gambir* extracts against MTA 48 h over seven days

**Time (day)**		***Uncaria gambir* extracts and MTA 48 h**								
		**1000 μg/ml Ug**	**500 μg/ml Ug**	**250 μg/ml Ug**	**125 μg/ml Ug**	**62.5 μg/ml Ug**	**31.25 μg/ml Ug**	**15.6 μg/ml Ug**	**7.8 μg/ml Ug**	**MTA 48 h**
1	FI (mean ± SD)	521.78 ± 4.09	467.76 ± 18.08	457.04 ± 32.80	459.38 ± 9.40	452.18 ± 16.69	450.78 ± 17.21	447.28 ± 28.90	446.48 ± 16.90	433.74 ± 15.13
	*p*-value	0.001	0.201	0.913	0.220	0.771	0.845	0.995	0.965	-
2	FI (mean ± SD)	592.10 ± 7.87	755.96 ± 57.09	769.24 ± 31.09	766.58 ± 16.13	774.22 ± 39.93	773.22 ± 13.29	781.44 ± 33.75	778.74 ± 22.16	746.7 ± 21.88
	*p*-value	< 0.001	1.000	0.949	0.466	0.940	0.537	0.721	0.536	-
3	FI (mean ± SD)	535.34 ± 10.07	866.08 ± 12.69	956.96 ± 42.35	986.56 ± 72.05	988.56 ± 37.94	1013.56 ± 21.29	1010.96 ± 30.11	1037.76 ± 33.76	1010.36 ± 73.17
	*p*-value	0.001	0.119	0.925	1.000	1.000	1.000	1.000	0.999	-
7	FI (mean ± SD)	644.42 ± 5.40	785.60 ± 23.02	1009.16 ± 21.38	1024.56 ± 7.40	1041.56 ± 5.59	1075.16 ± 20.33	1085.36 ± 16.95	1089.76 ± 48.84	775.24 ± 49.66
	*p*-value	0.046	1.000	0.002	0.004	0.003	0.001	0.001	< 0.001	-

*FI* Fluorescence intensity, *SD* Standard deviation, *Ug Uncaria gambir*, *MTA* Mineral trioxide aggregate

Multiple comparisons with Games-Howell post hoc test for comparison of *Uncaria gambir* extracts with MTA 48 h, *p* is statistically significant at α = 0.05

### Live and dead cell assay

The Live and Dead Cell assay was conducted on days 1, 2, 3 and 7 to assess the viability of MC3T3-E1 cells following exposure to various test materials and control solutions. The assay stained the viable cells with green fluorescence and dead cells with red fluorescence which can be analysed under fluorescent microscopy.

The *Uncaria gambir* extract below 250 µg/mL, 0.5% DMSO and negative control showed an abundance of green fluorescence viable cells which increased in intensification and density over the study period. Samples on day 7 showed a scanty amount of red fluorescence cells, interposed between the heavily-populated green fluorescence cells. Both MTA 4h and positive control showed similar presentation where the presence of multiple red fluorescent dead cells was observed throughout the 7 days.

**Fig. 3:**
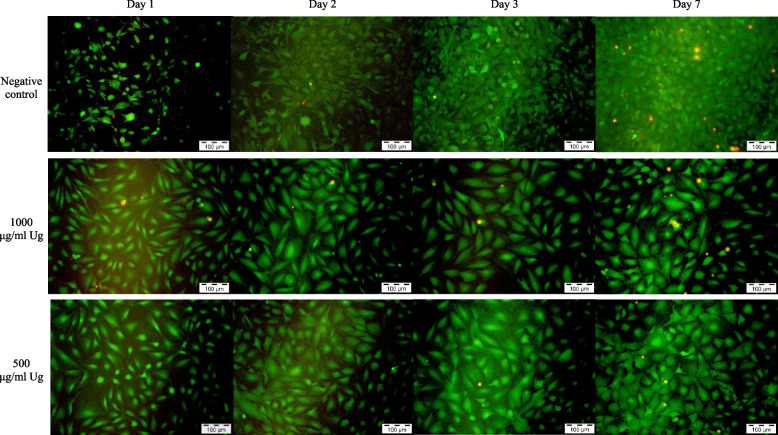
Live and Dead Cell analysis of MC3T3-E1 cells under the fluorescence microscope (x10) (*Uncaria gambir* and negative control)

**Fig. 4:**
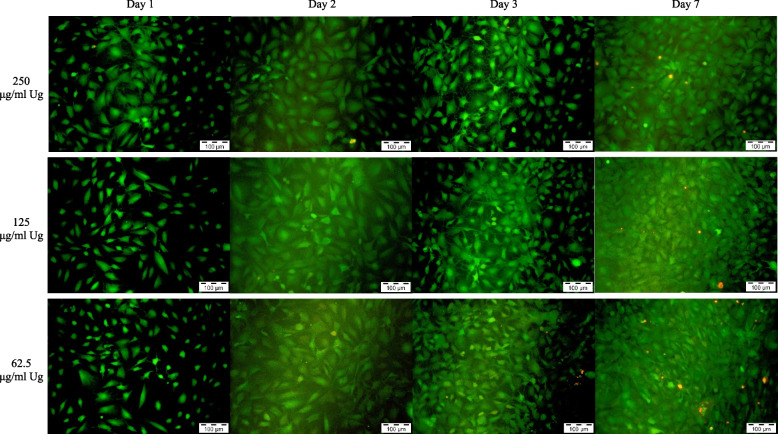
Live and Dead Cell analysis of MC3T3-E1 cells under the fluorescence microscope (x10) (*Uncaria gambir*)

**Fig. 5:**
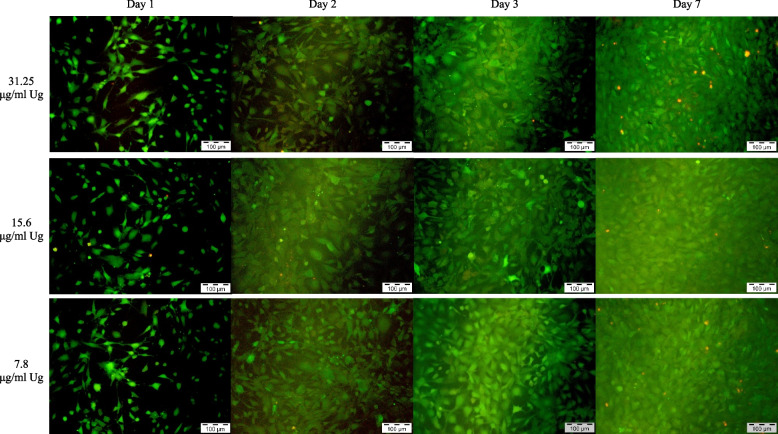
Live and Dead Cell analysis of MC3T3-E1 cells under the fluorescence microscope (x10) (*Uncaria gambir*)

**Fig. 6:**
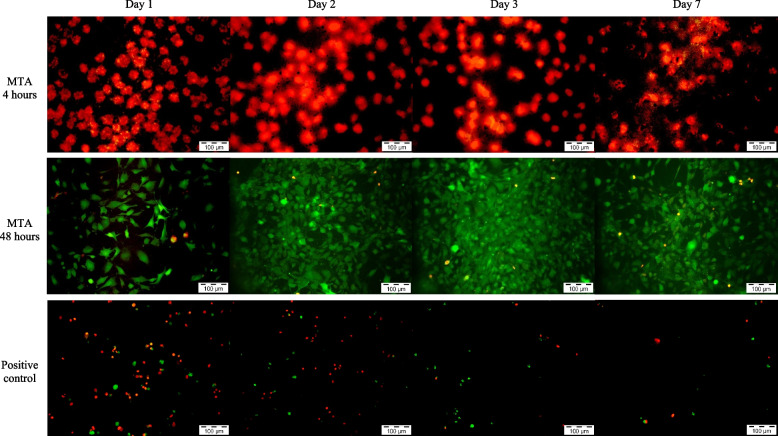
Live and Dead Cell analysis of MC3T3-E1 cells under the fluorescence microscope (x10) (MTA and positive control)

**Fig. 7:**
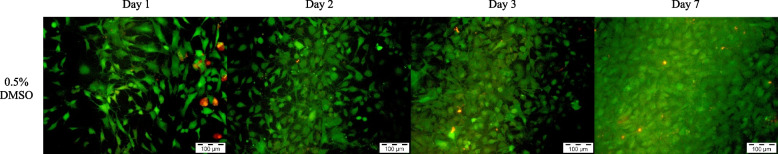
Live and Dead Cell analysis of MC3T3-E1 cells under the fluorescence microscope (x10) (0.5% DMSO)

*Uncaria gambir* extract at 1000 µg/mL demonstrated a higher density of green fluorescence cells as compared to negative control on day 1. However, the number of green fluorescence cells increased scarcely from day 2 onwards with distinctively lesser cell density in contrast to the negative control. Both MTA 48h and *Uncaria gambir* extract at 500 µg/mL showed a similar density of green fluorescence cells as compared to negative control up to day 3 before a reduction in intensification and density of green fluorescence cells were observed on day 7 (Figs. 3, 4, 5, 6 and 7).

### TEM analysis

**Fig. 8:**
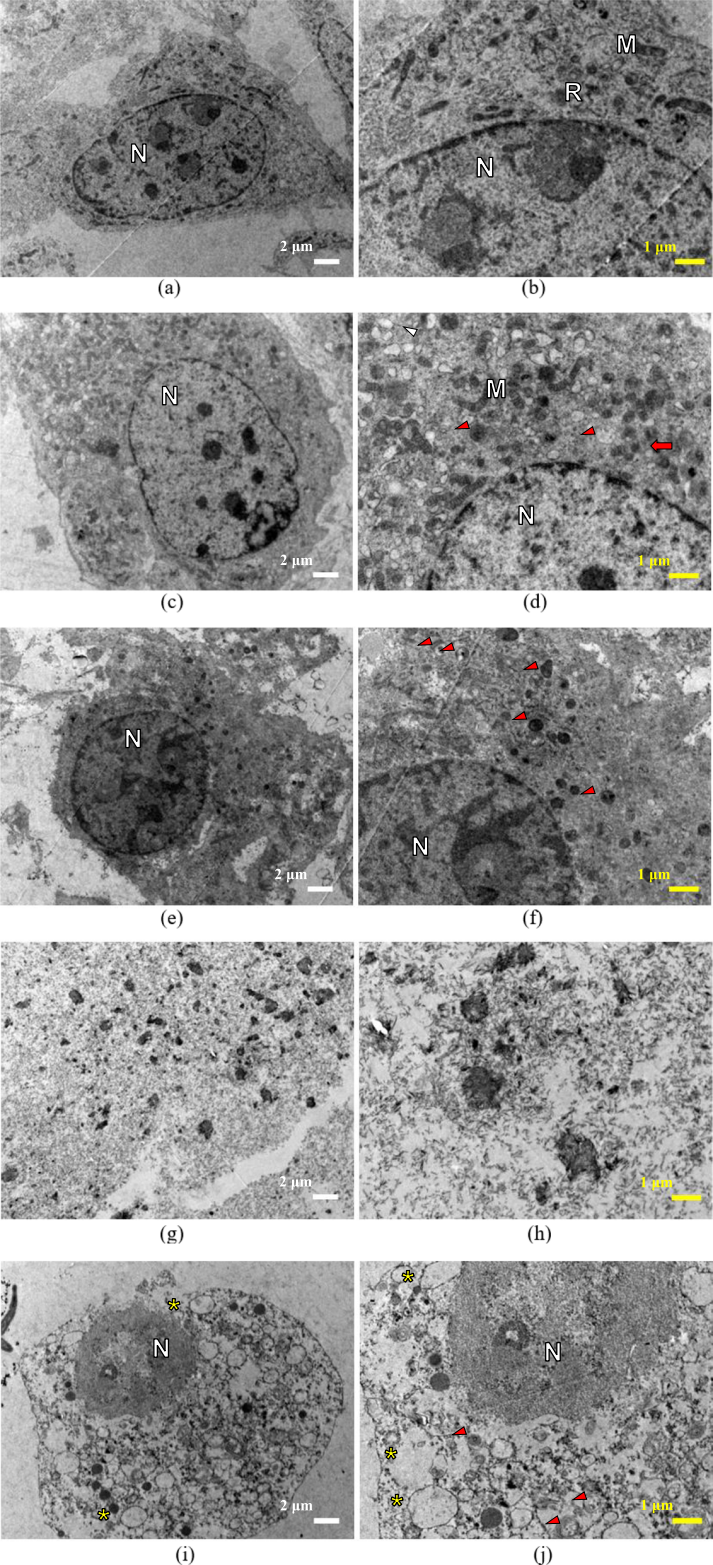
TEM images of MC3T3-E1 cells cultured in the following medium on day 1. **a** negative control (culture medium); (**b**) an enlargement of the images of (**a**). **c** 500 μg/ml *Uncaria gambir* extract; (**d**) an enlargement of the images of (**c**). **e** MTA eluate with 48 h setting time; (**f**) an enlargement of the images of (**e**). **g** MTA eluate with 4 h setting time; (**h**) an enlargement of the images of (**g**). The nuclear membrane and organelles were degraded whereas the nuclear contents and cytoplasmic components were dispersed into extracellular space. (**i**) 10% DMSO (positive control); (**j**) an enlargement of the images of (**i**). Degradation of the nuclear membrane and nuclear chromatin, the permeabilisation of the cell membrane and cytoplasmic vacuolation (yellow asterisk) were observed. The white bar represents 2 μm (magnification x4000); the yellow bar represents 1 μm (magnification x6000). N: nucleus, R: rough endoplasmic reticulum, M: mitochondria, swollen mitochondria (red arrow), autolysosomes and autophagic vesicles (red arrowhead)

On day 1, the cells exposed to negative control showed well-organised mitochondria and rough endoplasmic reticulum with the presence of ribosomes, intact nucleus and cell membrane and a normally distributed heterochromatin and euchromatin can be seen within the nucleus (Fig. 8a and b). The microscopic images of cells treated with 500 µg/mL of *Uncaria gambir* extract displayed intact nuclear membrane and nuclear content. The Golgi apparatus and rough endoplasmic reticulum can be seen within the cell’s cytoplasm. In addition, swelling of some mitochondria and autolysosomes was also observed (Fig. 8c and d). A similar presentation was observed in cells exposed to MTA 48h, with well-organised organelles and intact nuclear membrane. However, the number of organelles was lesser in contrast to the negative control and *Uncaria gambir* at 500 µg/mL concentration respectively. Autolysosomes and autophagic vesicles were seen scattered within the cytoplasm of the cells (Fig. 8e and f). Contrastingly, well-defined cell structure was absent in cells co-cultured with MTA 4h and the positive control. Degradation of the nuclear membrane and degeneration of mitochondria and other organelles were observed, with the dispersion of the nuclear content and cytoplasmic component into the extracellular space (Fig. 8g-j).

**Fig. 9:**
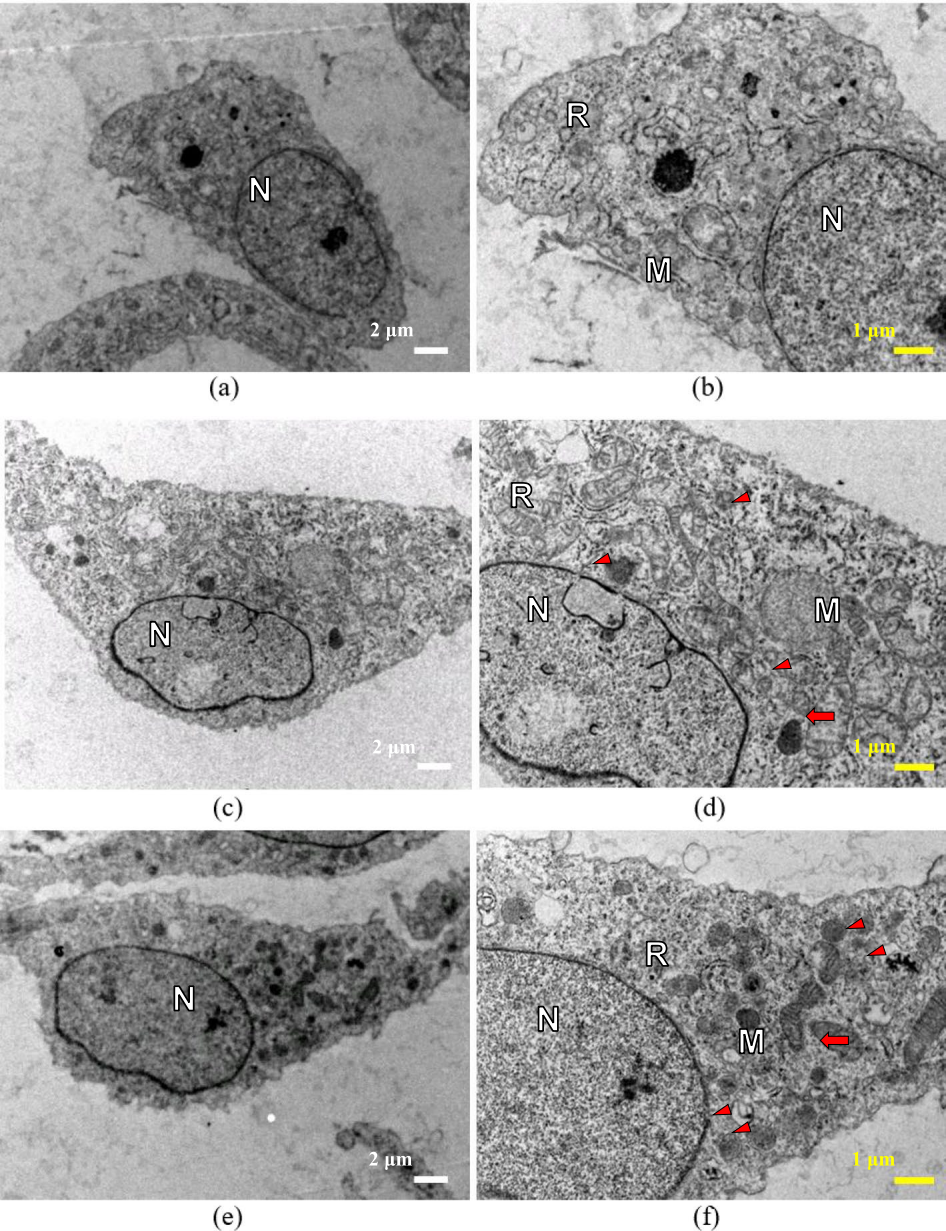
TEM images of MC3T3-E1 cells cultured in the following medium on day 7. **a** negative control (culture medium); (**b**) an enlargement of the images of (**a**). **c** 500 μg/ml *Uncaria gambir* extract; (**d**) an enlargement of the images of (**c**). **e** MTA eluate with 48 h setting time; (**f**) an enlargement of the images of (**e**). The white bar represents 2 μm (magnification x4000); the yellow bar represents 1 μm (magnification x6000). N: nucleus, R: rough endoplasmic reticulum, M: mitochondria, swollen mitochondria (red arrow), autolysosomes and autophagic vesicles (red arrowhead)

The cells exposed to negative control on day 7 displayed a similar presentation as day 1, with an increased number of organelles within the cell’s cytoplasm (Fig. 9a and b). The microscopic images of cells treated with *Uncaria gambir* extract at 500 µg/mL and MTA eluates at 48h were comparable. Both cells exhibited intact nuclear membrane and organelle, with fine chromatin. Some of the mitochondria were seen enlarged and swollen, while others maintained their integrity. Further, more autophagic vesicles were observed in cells exposed to MTA 48h as compared to *Uncaria gambir* extract at 500 µg/mL concentration (Fig. 9c-f).

## Discussion

The current study was our preliminary work in the development of a novel natural-based pulpotomy medicament based on the *Uncaria gambir* extract. The cytotoxic study of new material is of paramount importance, mainly in determining the safety concentration of the material that exerts the maximum therapeutic effects and minimises the potential cellular damage that might affect cellular function which may lead to harmful side effects. Comparative evaluation with MTA as the gold standard pulpotomy medicament will give valuable insights into the investigation of the potential usage of *Uncaria gambir* as an alternative medicament for pulpotomy. MTA was prepared as eluates, where the leachable content from the set material is collected in the culture media. This will reflect the actual scenario intra-orally as the material was under constant challenge by saliva and dentinal fluids [26, 27]. Two formulations of MTA eluates were used in this study, MTA with 4- and 48-h setting times respectively. The 4 h setting time is the primary setting time based on the manufacturer’s recommendation, while MTA with 48 h setting time is the complete setting time as proposed by other studies in the literature [19, 27].

The cytotoxicity of the test materials was in the following order: MTA 4h > *Uncaria gambir* 1000 µg/mL > *Uncaria gambir* 500 µg/mL = MTA 48h > *Uncaria gambir* 250 µg/mL and below. This was reflected by the reduction in fluorescence intensity and cell density measured through Alamar Blue and Live and Dead Cell assay respectively, which were consistent with each other. The outcome of the cytotoxic assays was further supported by the TEM analysis, where an increased amount of autophagic vesicles and cytoplasmic autolysosomes were detected in some cells on day 7 following exposure to MTA 48h and *Uncaria gambir* at 500 µg/mL. The presence of autophagic vesicles and autolysosomes is associated with the deranged and degradation of cellular compartment including the proteins and organelles that maintains cell survival [28]. This explained the reduction in cell viability of M3CT3-E1 cells over time after being exposed to both materials.

A statistically significant difference was observed for the cells treated with MTA 48h and *Uncaria gambir* extract at 500 µg/mL as compared to the negative control solutions for days 7 and days 3 and 7 respectively. Nevertheless, the significant difference should be interpreted with caution. The difference was significant due to the decreased cell viability over time following treatment with MTA 48h and 500 µg/mL *Uncaria gambir* extract, whereas the percentage of cell viability of negative control remained at 100% throughout the study period. Thus, since the mean percentage of cell viability on day 7 exceeded the 70% threshold as outlined by ISO standard [22], the materials are regarded as non-cytotoxic toward the MC3T3-E1 cells.

*Uncaria gambir* extract at a concentration below 250 µg/mL was constantly not cytotoxic to the cells, with cell viability of more than 90% during the 7 days exposure period, similar to those depicted by negative control. Interestingly, the Live and Dead Cell assay of day 7 samples showed some red-stained dead cells ‘floating’ against the background of overly-populated green cells for both materials. We believe the findings were due to the death of some cells following competition for survival and not because of the cytotoxic nature of the material. As the cell growth increases over time, the overly populated cells start to compete with each other for the depleting amount of culture media.

The results of our study were in agreement with other studies reported in the literature. The cytotoxicity of MTA has been described by Kim et al. [20] and Bueno et al. [8]. Both of the authors observed severe cytotoxic and inflammatory effects of ProRoot® MTA on the cells tested. The cytotoxic effects of MTA during its setting phase were believed due to the leaching out of the component from the material itself, which can directly affect the cell. In addition, MTA releases hydroxyl ions into its surroundings as part of its antibacterial mechanism [29], which in turn can alter the pH of the local environment and impair the plasma membrane and disrupt the cell’s enzymatic function [8]. On the other hand, Anggraini et al. [10] and Nur Sazwi et al. [17] have investigated the cytotoxic effects of *Uncaria gambir* extract. The former authors evaluated the cytotoxic effect of *Uncaria gambir* on the intestinal epithelial cell line. The experimental gambir was biocompatible towards the cell lines with cell viability of over 93% at a concentration below 200 µg/mL. A higher concentration was reported by the latter author, where the commercially prepared *Uncaria gambir* at a concentration between 250 to 500 µg/mL was found to be non-cytotoxic when tested against human gingival fibroblast cells.

We postulated that the presence of the active component within the extract of *Uncaria gambir* is the main reason for the cytotoxic effects exerted by the extract at a concentration beyond 500 µg/mL. The present study is our preliminary investigation of the cytotoxic potential of *Uncaria gambir* extract and we did not perform any compound analysis at this stage yet. However, in the literature it was reported that catechin is the primary flavonoid found within the *Uncaria gambir* extract, making up to 80% of its total flavonoid content [10], alongside quercetin, epicatechin, and procyanidin [17]. At high concentrations, catechin and other flavonoid compounds have been reported to cause cytotoxic effects against the cells in vitro. The effects of catechin and its gallic acid derivatives on the renal tubular cells were investigated by Miyamoto et al. [30] in vitro. The high concentration of catechins led to the cell’s DNA fragmentation and growth inhibition when viewed under the microscope, reflecting the cytotoxic nature of the compound at that particular concentration. Similarly, quercetin at high concentration evokes a reduction in cell viability and survival as opposed to the cell proliferation effects stimulated by the compound at low concentration [31]. Further, Kocyigit et al. [32] concluded that the cytotoxic effects of their experimental flavonoids were exhibited in a dose-dependent manner.

It has been suggested that catechin can exert both antioxidant and pro-oxidant activities at different concentrations. While the antioxidant activity is associated with and exerted at low and optimum concentrations, the pro-oxidant effect of the compound has been observed at high concentrations [31–33]. The pro-oxidant activity is related to the generation of reactive oxygen species and peroxidase-induced oxidation that increased in number within the cells. Later, this leads to the autophagic vacuolization of the mitochondria and other organelles [34], similar to those observed in our TEM analysis. This explains the presence of scattered vesicles and vacuoles within the cytoplasm of the cells. In addition, a higher concentration of catechin beyond its cytotoxic threshold leads to the partitioning of the lipid bilayer of the cell membrane and disrupts its integrity through the lateral expansion mechanism [35, 36] and later causes cell death.

*Uncaria gambir* extract at 500 µg/mL showed a similar pattern of declination in terms of cell viability and fluorescence intensity as MTA 48h. The two-way mixed ANOVA showed no significant difference between the extract at 500 µg/mL and the gold standard pulpotomy material. The extract at a concentration below 250 µg/mL showed better cell proliferation and viability as opposed to MTA 48h. Further, MTA 48h showed a steeper drop in cell viability from day 3 to day 7. These changes were also apparent in the Live and Dead Cell assay where the density of the green fluorescence viable cells reduced markedly on day 7 as compared to the first three days of exposure. Lesser organelle and more autophagic vesicles and autolysosomes were observed in a cell exposed to MTA 48h as compared to the *Uncaria gambir* at 500 µg/mL when viewed under TEM, especially on the samples of day 7.

Our study has confirmed the non-cytotoxic concentration of *Uncaria gambir* extract which is below 500 µg/mL The effects were comparable to those exerted by MTA. Since MTA has been used widely as a pulpotomy medicament with a good success rate, *Uncaria gambir* extract offers huge potential which can be explored further as an alternative pulpotomy medicament.

Nevertheless, there are some limitations in the present study. This in vitro study was still in its preliminary stage and further investigation on the antibacterial, anti-inflammatory and antioxidant effects of *Uncaria gambir* extract at 500 µg/mL concentration should be conducted in the near future. The potential antibacterial effects of the extract against oral microflora will further justify its usage as a pulpotomy medicament. To the best of our knowledge, catechin is the main secondary metabolite found in *Uncaria gambi*r extract. However, other flavonoid compounds are present within the extract. Analysis of the compound, for example using High-Performance Liquid Chromatography (HPLC), will give a better insight into which compound might influence the abovementioned effects, either in combination or in solitary. The exact mechanism of action of the extract also can be elucidated, adding more value to the development of medicament for pulpotomy based on the extract of *Uncaria gambir.*

In addition, we used MC3T3-E1 cells, which are derived from an animal source. Human dental pulp cells can be considered in future studies, as they represent the actual cells that will be in contact with the material clinically. The cellular reaction mainly the immune response and cell metabolism between animal and human cells might be different [26], hence the usage of human dental pulp cells may give better insights into the cytotoxic evaluation of the material. The extraction of *Uncaria gambir* extract in the present study used a conventional maceration extraction method, which requires a high volume of solvent and extraction time. Supercritical fluid extraction, pressurised liquid extraction and ultrasound-assisted extraction are among the new methods that can be utilised in the future, due to their being rapid, having higher selectivity towards the desired bioactive compounds, able to increase the extraction yield and environment-friendly [37].

## Conclusion

MTA 48h and *Uncaria gambir* extract with concentrations below 500 µg/mL were biocompatible and non-cytotoxic towards MC3T3-E1 cell lines over time as depicted by the viable cells of over 70%, observed via the in vitro cytotoxic tests. *Uncaria gambir* extracts at different concentrations achieved higher cell viability and exhibited less cellular toxicity as compared to MTA 4h. Moreover, the biocompatibility of *Uncaria gambir* extracts with concentrations below 500 µg/mL was comparable to MTA 48h which was the gold standard pulpotomy medicament.

## Data Availability

The datasets used and/or analysed during the current study are available from the corresponding author upon reasonable request.

## Abbreviations

ANOVA: Analysis of Variance
CO_2_: Carbon dioxide
DMSO: dimethyl sulfoxide
EDTA: ethylenediaminetetraacetic acid
HPLC: High-Performance Liquid Chromatography
IHC: immunohistochemistry
ISO: International Standard Organisation
MTA: mineral trioxide aggregate
TEM: transmission electron microscope
